# Systematic Comparison of Four Methods for Detection of Carbapenemase-Producing *Enterobacterales* Directly from Blood Cultures

**DOI:** 10.1128/JCM.00709-19

**Published:** 2019-10-23

**Authors:** Maria Meier, Axel Hamprecht

**Affiliations:** a Institute for Medical Microbiology, Immunology and Hygiene, University Hospital of Cologne, Cologne, Germany; b DZIF (German Centre for Infection Research), Bonn-Cologne, Germany; Johns Hopkins University School of Medicine

**Keywords:** carbapenemase, Carba NP, blood stream infection, NDM, OXA-48, KPC, VIM, β-Carba, CIM, CPE, colorimetric

## Abstract

Early identification of infections caused by carbapenemase-producing *Enterobacterales* (CPE) can help to optimize patient treatment and improve outcome. In this study, protocols for rapid detection of carbapenemase production directly from positive blood cultures were developed applying a concentration and hemolysis step before a test for carbapenemase production was performed. Four different methods (three modified colorimetric assays [β-Carba, bcCarba NP, and NeoRapid Carb] and a variation of the carbapenem inactivation method [CIM] test with blood cultures [bcCIM]) were assessed on blood cultures spiked with 185 different molecularly characterized *Enterobacterales* isolates. The challenge collection included 81 carbapenemase-negative isolates and 104 CPEs (OXA-48 [*n* = 25], NDM [*n* = 20], KPC [*n* = 18], VIM [*n* = 25], GIM [*n* = 5], OXA-48-like [*n* = 9], and OXA-48-like plus NDM [*n* = 2]). The sensitivity/specificity was 99.0%/95.1% for bcCarba NP, 99.0%/91.4% for NeoRapid Carb, 100%/95.1% for β-Carba and 100%/100% for bcCIM. Weakly hydrolyzing carbapenemases (e.g., OXA-48-like) were also well detected by the assays. The time to result was 20 to 45 min for β-Carba, 2 to 3 h for bcCarba NP, 2.5 to 2 h for NeoRapid Carb, and 18 to 24 h for bcCIM. In conclusion, all assays demonstrated good detection of CPE. The protocols can be easily implemented in any clinical microbiology laboratory and could help to optimize therapy early in bloodstream infections by CPE.

## Abstract (precis)

Early identification of infections caused by carbapenemase-producing *Enterobacterales* (CPE) can help to optimize patient treatment and improve outcome. In this study, protocols for rapid detection of carbapenemase production directly from positive blood cultures were developed applying a concentration and hemolysis step before a test for carbapenemase production was performed.

## INTRODUCTION

Blood stream infections (BSI) with carbapenemase-producing *Enterobacterales* (CPE) are associated with higher mortality and increased cost and length of hospital stay (1). The prompt initiation of appropriate antibiotic therapy can improve patient management and clinical outcome; the rapid detection of CPE is therefore of utmost importance. Standard methods used today (e.g., conventional disk diffusion testing or other susceptibility techniques) typically require 16 to 20 h after a blood culture has become positive to detect carbapenem resistance. In the case of carbapenem resistance, further confirmatory tests have to be performed for the detection of a carbapenemase, which take additional time, e.g., colorimetric tests, PCR, immunochromatographic tests (ICTs), or the carbapenem inactivation method (CIM) or modified CIM (mCIM) (2–5). Different assays can shorten the time for the detection of CPE from BSI, including, nucleic acid-based techniques, matrix-assisted laser desorption ionization–time of flight (MALDI-TOF) analysis, and others (6–9). However, for most tests, significant hands-on time or additional incubation steps on agar or in broth are required. Furthermore, some tests (e.g., PCR and ICT) only target a subset of carbapenemases (e.g., the four most common: OXA-48, KPC, NDM, and VIM).

The advantage of colorimetric assays (e.g., Carba NP test) is the broader detection of carbapenemase activity than for most available PCRs or ICTs. Colorimetric assays contain a carbapenem which is hydrolyzed by a carbapenemase and subsequently leads to a pH shift resulting in a color change of an indicator (e.g., bromothymol blue or phenol red) (2, 10). Besides Carba NP, several commercial colorimetric tests are available, e.g., Rapidec Carba NP, NeoRapid CARB, or β-CARBA (11–15). These tests have been validated for the use from solid media; additionally, protocols for the direct detection of CPE from blood cultures have been proposed. However, usually, an additional culture on solid or liquid medium (2 to 4 h) is necessary, which is followed by lysis and incubation in the presence of a carbapenem and the pH indicator, resulting in a long hands-on time and a long time to result (6). Most studies have investigated a single assay only, and few data are available on the comparison of different phenotypic tests for CPE detection from blood cultures (16).

In the present study, we developed rapid protocols for the detection of carbapenemases from positive blood culture bottles and systematically compared the performance of four different tests on a collection of 185 *Enterobacterales* isolates.

## MATERIALS AND METHODS

### Bacterial isolates.

A total of 185 nonduplicate *Enterobacterales* isolates from different years, wards, and centers were included in the analysis (Table 1). One hundred four isolates were carbapenemase producers which had been characterized in previous studies (17–20): OXA-48 (*n* = 25), NDM (*n* = 20), KPC (*n* = 18), VIM (*n* = 25), GIM (*n* = 5), OXA-48-like (*n* = 9), OXA-48-like plus NDM (*n* = 2). Additionally, 81 strains served as negative controls, most of them produced extended-spectrum β-lactamase (ESBL); additionally, two quality control strains without beta-lactamase activity were included (Escherichia coli ATCC 25922 and E. coli J53).

**TABLE 1:** Isolates included in the study

Carbapenemase production	No. of isolates					
	K. pneumoniae	E. coli	Enterobacter cloacae	Citrobacter freundii	Others^*a*^	All species
Carbapenemase positive	39	26	13	19	7	104
OXA-48-like	12	18	2	2		
OXA-48	7	14	2	2		
OXA-162	1	1				
OXA-181	1	1				
OXA-204	1					
OXA-232	1	1				
OXA-244	1	1				
KPC	16			1	1	
KPC-2	15			1	1	
KPC-3	1					
NDM	8	5	3		4	
NDM-1	8	4	3		4	
NDM-7		1				
VIM	2	2	4	16	1	
VIM-1	2	2	2	12		
VIM-2				3		
VIM-4			1	1		
VIM-27			1			
VIM-39					1	
GIM			4		1	
GIM-1			4		1	
Multiple carbapenemases						
NDM-1/OXA-232	1					
NDM-5/OXA-181		1				
Carbapenemase negative	18	55	3	2	3	81
Total	57	81	16	21	10	185

a Others includes K. oxytoca, Citrobacter braakii, Enterobacter asburiae, Raoultella ornithinolytica, Proteus mirabilis, Providencia stuartii, and Serratia marcescens.

All strains were analyzed phenotypically for carbapenemases using meropenem disks in combination with the inhibitors EDTA, boronic acid, and cloxacillin (Liofilchem, Roseto degli Abruzzi, Italy). Additionally, all isolates with elevated MICs for ertapenem, imipenem, or meropenem were assessed by PCR for the presence of *bla*_OXA-48-like_, *bla*_VIM_, *bla*_NDM_, *bla*_GIM_, *bla*_IMP_, and *bla*_KPC_ (17–19, 21, 22). Carbapenemase-negative isolates were characterized for ESBLs using the CLSI combination disk test. Additionally, isolates were assessed by microarray Check-MDR CT101 (Check-Points, Wageningen, Netherlands) for production of *bla*_CTX-M-1 group_, *bla*_CTX-M-2 group_, *bla*_CTX-M-9 group_, *bla*_TEM_, and *bla*_SHV_. Isolates which were positive for *bla*_CTX-M_ were further characterized by sequencing the open reading frame as previously described (23) using primers listed in Table S4 in the supplemental material.

### Inoculation and processing of blood cultures.

A bacterial suspension equivalent to McFarland 0.5 was prepared in a 0.85% natrium-chloride solution from bacterial colonies grown on Columbia blood agar and diluted 1:1,000. Subsequently, 10 μl of this diluted suspension was mixed with 5 ml human blood from healthy volunteers for a final inoculum of ∼300 CFU/ml and inoculated into BD Bactec Plus Aerobic blood culture bottles (BD, Heidelberg, Germany). Bottles were incubated in a Bactec FX automated blood culture system (BD); after blood cultures were flagged as positive, 7 ml blood culture fluid was drawn and used for the different assays. Additionally, all blood cultures were tested for purity by inoculating 100 μl of blood culture fluid onto sheep blood agar.

### Evaluation of hemolysis protocols.

The first step of developing rapid test protocols for the analysis of blood cultures was the evaluation of a suitable hemolytic agent for each test method. Therefore, the following hemolytic agents were assessed with 10 test isolates: saponin (Sigma-Aldrich, Steinheim, Germany), sodium dodecyl sulfate (SDS; AppliChem, Darmstadt, Germany), ammonium-chloride potassium lysis buffer (ACK; consisting of 150 mM KHCO_3_ [Carl Roth, Karlsruhe, Germany] abd 10 mM NH_4_Cl [Sigma-Aldrich]), and Triton X (Sigma-Aldrich). The impacts of the hemolysis protocols on results and readability were analyzed. Additionally, different sample volumes were compared to achieve the overall best sensitivity without giving rise to false-positive results. For the β-CARBA test, 10% SDS provided complete hemolysis without interference with the test. For the Carba NP test and NeoRapid CARB, 5% saponin showed the best results for hemolysis with minimal interference and was therefore selected for the study. Triton-X and ACK lysis buffer resulted in less complete hemolysis and/or gave rise to false-positive results (data not shown). After blood culture fluid was drawn for the different protocols and erythrocytes were lysed, the bacterial pellet was washed with water and phosphate-buffered saline (PBS; pH 7.4) for all protocols to optimize readability and pH values.

### bcCarba NP test.

A modified procedure of Carba NP using blood cultures (bcCarba NP) was developed that did not require an additional incubation in brain heart infusion (BHI) broth as previously described for this assay (2, 6). For bcCarba NP, 2 × 1 ml blood culture fluid was transferred to 1.5-ml reaction tubes and mixed with 200 μl of saponin 5% (Sigma-Aldrich Chemie, Munich, Germany) to lyse the blood cells. After incubating at room temperature for 5 min, the samples were centrifuged and supernatants discarded (all centrifugation steps were undertaken at 13,000 × *g* for 1 min). The bacterial pellets were washed with 1 ml distilled water and 1 ml PBS. Subsequently, 50 μl NaCl 0.85% and 50 μl bacterial protein extraction reagent (B-PERII; Thermo Fisher Scientific, Duisburg, Germany) were added to lyse the bacterial cells. After 30 min of incubation at room temperature, 100 μl test solution without imipenem (negative control) was added to the first reaction tube and 100 μl with imipenem (positive control) was added to the other reaction tube. During incubation at 37°C for a maximum of 2 h, any color change of the positive control from red to orange or yellow, while the negative control remained red, was interpreted as positive. Test solutions were prepared according to the methods described by Nordmann et al. (2).

### β-CARBA test.

For the β-Carba test (Bio-Rad Laboratories, Munich, Germany), 2 ml of the positive blood culture was mixed with 200 μl of 10% SDS (AppliChem, Darmstadt, Germany) and incubated at room temperature for 5 min to lyse erythrocytes. The sample was centrifuged subsequently, and the supernatant was discarded. The bacterial pellet was washed with 1 ml distilled water and 1 ml PBS. After centrifugation and discarding the supernatant, the bacterial pellet was subjected to the β-Carba test as recommended by the manufacturer. Briefly, the pellet was mixed with 40 μl of R1 reagent and 40 μl of R2. After incubating at 37°C for 30 min, the results were read. Any color change within these 30 min from yellow to orange, red, or purple was interpreted as positive.

### NeoRapid CARB.

For the NeoRapid CARB (Rosco, Taastrup, Denmark), 1 ml of the blood culture fluid was lysed with 5% saponin as described above for the Carba NP test. After washing, 150 μl 0.85% NaCl and 50 μl B-PERII were added and incubated at room temperature for 30 min; 100 μl of this mixture was transferred to a second tube. Then, 100 μl 0.85% NaCl was added to both tubes. The tablet containing imipenem was added to one tube (positive control), the tablet without imipenem was added to the other (negative control). During incubation at 37°C for 60 to 90 min, any color change of the positive control from red to orange or yellow, while the negative control remained red, was interpreted as positive.

### bcCIM.

For the blood culture CIM (bcCIM) test, 10 μl of 10 mM ZnSO_4_ was added to 1 ml of the blood culture fluid. A 10-μg meropenem disk (Oxoid, Wesel, Germany) was immersed in the fluid and incubated at 37°C for 2 h. After incubation, the meropenem disk was removed and placed on a Muller-Hinton agar (MHA) plate which had been inoculated with a suspension of E. coli ATCC 25922 (0.5 McFarland). The plate was incubated at 37°C for 16 to 22 h. Subsequently, the inhibition zone was measured and classified as follows: >19 mm, negative; 15 to 19 mm, indeterminate; <15 mm, positive for carbapenemase production (4).

## RESULTS

**FIG 1:**
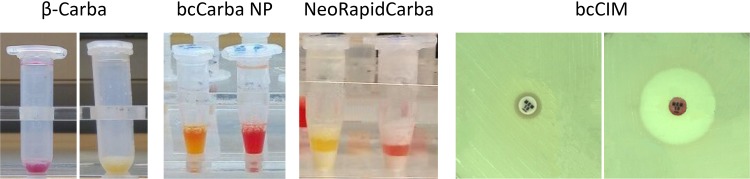
Test results with β-Carba, bcCarba NP, NeoRapid Carba, and bcCIM. A positive result for each test is shown on the left, with a negative result on the right.

With the new protocols that include a lysis/concentration step, good and rapid results were achieved. Carbapenemase production was detected by color change for β-Carba, from yellow to either orange, red, or purple, and from red to red/orange or yellow for bcCarba NP and the NeoRapid CARB kit (Fig. 1). β-Carba detected all carbapenemases, resulting in a sensitivity of 100% and specificity of 95.1% (Table 2). There were no differences between species and carbapenemases; also, weakly hydrolyzing enzymes were easily interpreted, including all OXA-48-like-producing isolates (*n* = 34) (see Table S1 in the supplemental material). bcCarba NP also gave good results, reaching 99.0% sensitivity and 95.2% specificity (Table 2). Only one of two OXA-244-expressing isolates produced a false-negative result (Table 3; see also Table S3). All other OXA-48-like-carbapenemases were detected. NeoRapid CARB detected all carbapenemases except one OXA-244, resulting in a sensitivity of 99.0% and specificity of 91.4% (Table 2).

**TABLE 2:** Comparison of the performance, hands on time, and costs of the different assays

Category	Value			
	β-Carba	bcCarba NP	NeoRapid CARB	bcCIM
Sensitivity (% [CI]^*a*^)	100 (96.5–100)	99.0 (97.2–100)	99.0 (97.2–100)	100 (96.5–100)
Specificity (% [CI])	95.1 (90.3–99.8)	95.1 (90.3–99.8)	91.4 (85.2–97.8)	100 (95.6–100)
Time to result	20–45 min	2–3 h	1.5–2 h	18–24 h
Cost per test ($)	∼6.20	∼1.90	∼2.40	∼0.85

a CI, 95% confidence interval.

**TABLE 3:** Performance of the assays stratified by carbapenemase variant

Carbapenemase	No. of isolates^*a*^							
	β-Carba		bcCarba NP		NeoRapid CARB		bcCIM	
	P	N	P	N	P	N	P	N
Ambler class D (*n* = 34)								
OXA-48 (*n* = 25)	25		25		25		25	
OXA-162 (*n* = 2)	2		2		2		2	
OXA-181 (*n* = 2)	2		2		2		2	
OXA-204 (*n* = 1)	1		1		1		1	
OXA-232 (*n* = 2)	2		2		2		2	
OXA-244 (*n* = 2)	2		1	1	1	1	2	
Total (*n* [%])	34 (100)		33 (97.1)	1 (2.9)	33 (97.1)	1 (2.9)	34 (100)	
Ambler class A (*n* = 18)								
KPC-2 (*n* = 17)	17		17		17		17	
KPC-3 (*n* = 1)	1		1		1		1	
Total (*n* [%])	18 (100)		18 (100)		18 (100)		18 (100)	
Ambler class B (*n* = 50)								
NDM-1 (*n* = 19)	19		19		19		19	
NDM-7 (*n* = 1)	1		1		1		1	
VIM-1 (*n* = 18)	18		18		18		18	
VIM-2 (*n* = 3)	3		3		3		3	
VIM-4 (*n* = 2)	2		2		2		2	
VIM-27 (*n* = 1)	1		1		1		1	
VIM-39 (*n* = 1)	1		1		1		1	
GIM-1 (*n* = 5)	5		5		5		5	
Total (*n* [%])	50 (100)		50 (100)		50 (100)		50 (100)	
OXA-48 plus NDM-1 (*n* = 2)	2		2		2		2	
Total (*n* [%])	2 (100)		2 (100)		2 (100)		2 (100)	
Total carbapenemase positive (*n* [%])	104 (100)		103 (99.0)	1 (1.0)	103 (99.0)	1 (1.0)	104 (100)	
No carbapenemases (*n* = 81)								
Total carbapenemase negative (*n* [%])	4 (4.9)	77 (95.1)	4 (4.9)	77 (95.1)	7 (8.6)	74 (91.4)		81 (100)

a P, positive result; N, negative result.

Most of the false-positive results with β-Carba, Carba NP, or NeoRapid CARB KIT were caused by Klebsiella oxytoca (three isolates) (Table S3).

For the adaptation of the CIM test for blood culture (bcCIM), different volumes were tested. The best results were achieved with 1 ml blood culture fluid. To improve the detection of metallo-beta-lactamases (MBL), supplementation with ZnSO_4_ was necessary. With this method, the presence of carbapenemases was detected reliably by assessing the inhibition zone (Fig. 1). All carbapenemases were detected by bcCIM and there were no false-positive results, resulting in a sensitivity of 100% and specificity of 100%.

## DISCUSSION

This study demonstrated that with the modification of phenotypic tests proposed, rapid and reliable detection of carbapenemase production can be achieved directly from positive blood cultures. With all methods, the most common carbapenemases could be detected within 20 to 45 min (β-Carba test), 1.5 to 2 h (NeoRapid CARB), 2 to 3 h (bcCarba NP), or 18 to 24 h (bcCIM). To achieve these results, it was necessary to concentrate bacteria and to hemolyze erythrocytes in order to avoid an impact on the color/reading of the colorimetric tests. Both the hemolysis procedure and inoculum were critical to obtain the observed sensitivities.

The β-Carba test achieved excellent results for the detection of the most common carbapenemases (VIM, KPC, NDM, OXA-48-like, and GIM), which exceeded those reported from other studies on this assay on bacterial colonies from agar plates (15, 17). The good performance of this assay from blood cultures could also be related to the medium and the inoculum, which have previously been shown to affect the performance of this test (15). Additionally, no isolates with IMI or GES production were included, which are not well detected with this test (17). Nevertheless, the β-Carba test achieved very reliable results in this study, with a sensitivity of 100% and specificity of 95.1%. These results are in line with results from the previous study on this assay from blood cultures (20); however, similar isolate collections were used in both studies, which could partly explain the comparable performance.

The detection of carbapenemases by bcCarba NP directly from positive blood cultures showed excellent sensitivity (99.0%) and specificity (95.1%). Compared to previous studies on Carba NP from blood cultures, bcCarba NP does not require additional cultivation on solid or in liquid media (6, 16), leading to a significant time reduction (2 to 3 h time to result instead of 5 to 6 h). The reliability of our protocol is similar to that in previous studies (6) but with a higher sensitivity in isolates producing OXA-48-like carbapenemases.

The modification of NeoRapid CARB allows the reliable detection of carbapenemases within a short time, without the need to prepare numerous different reagents, as for the “homemade” Carba NP/bcCarba NP test. Our protocol differed from that provided by the manufacturer by the use of saponin instead of Triton X-100 for hemolysis, because the protocol provided by the manufacturer gave rise to false-negative results among our OXA-48-producing isolates (data not shown). The specificity of the NeoRapid CARB was slightly lower than for the other tests. However, other studies show similar results on isolates grown on solid medium, with sensitivity and specificity values ranging from 89% to 100% and 70% to 100%, respectively (11, 13, 14).

We also tested another commercial version of Carba NP (Rapidec Carba NP). However, with positive blood cultures as a matrix, no reliable results were achieved and the assay was not further investigated.

The overall better performance of the β-Carba test than of the two other colorimetric tests could be the result of the higher volume/inoculum which could be used with this assay (2 ml) or the different hemolysis reagent. For the other assays, a volume of 2 ml gave rise to false-positive results and impaired readability; therefore, 0.5 ml (NeoRapid CARB) or 1 ml (bcCarba NP) was employed. Additionally, different hemolysis reagents had to be used, since some reagents affected readability and gave rise to false-positive or false-negative results, e.g., when SDS was used with bcCarba NP or NeoRapid Carb. We observed that K. oxytoca isolates gave rise to false-positive results in the three colorimetric tests from blood culture fluid (see Table S3 in the supplemental material). These isolates were correctly identified as carbapenemase negative when the tests were performed from Columbia blood agar, and the false-positive result is therefore likely caused by the blood culture medium or the hemolysis procedure. It is difficult to reliably identify the cause of the false-positive results, as three different tests (β-Carba test, bcCarba NP, and NeoRapid CARB) were affected; additionally, two different hemolytic agents (SDS for β-Carba and saponin for bcCarba NP and NeoRapid CARB) and test principles were involved. The three K. oxytoca isolates also produced different β-lactamases (Hyper K1, CTX-M-9, and CTX-M-3/TEM-1). Therefore, the false-positive results are linked to the species rather than the β-lactamase. Noel et al. also describe false-positive results for K. oxytoca and the β-Carba test (14). Among our collection, K. oxytoca was overrepresented compared to the epidemiology of Gram-negative bloodstream infections. Likely, this problem will occur less frequently in clinical routine. A solution could be to perform species identification from blood cultures, at least when a positive result is achieved. This can ideally be done by MALDI-TOF, using the same pellet as for the carbapenemase assays (24). This has the advantage that the species identification provides additional information for the selection of the most appropriate therapy.

The false-negative results for bcCarba NP (*n* = 1) and NeoRapid CARB (*n* = 1) were both recorded in OXA-244-producing isolates (Table S3). The tests were repeated both from a freshly inoculated blood culture and from bacteria harvested from Columbia blood agar, which also led to the same results. Therefore, it is unlikely that the hemolysis protocol caused the false-negative results. OXA-244 belongs to the group of OXA-48-like carbapenemases which are weakly hydrolyzing enzymes, which likely explains these results. A lower sensitivity of Carba NP for OXA-48-like carbapenemases (91.3% versus 100% for other carbapenemases) was described previously (6).

The CIM test and the modified version (mCIM) are based on the inactivation of meropenem by carbapenemases (3, 4) and are recommended by CLSI as a phenotypic method for carbapenemase detection on isolates grown on solid media. In the present study, a protocol for the use from blood cultures (bcCIM) was developed. With bcCIM, all carbapenemase-producing isolates were reliably identified, and no carbapenemase-negative strain led to false-positive results. Our results are in line with studies using samples from solid media (3, 4). Using blood culture samples, MBLs were only partly detected. To improve the detection of MBLs, zinc sulfate was added to the blood samples, leading to a sensitivity of 100% including MBLs. An improved sensitivity of mCIM with zinc supplementation (zCIM) was also recently reported for isolates grown on solid media (17). Despite the high sensitivity, specificity, and low costs, bcCIM has the disadvantage of a long time to result and therefore cannot be considered a rapid test. It has to be further investigated if similar results can be reached by a shorter incubation time (e.g., 4 to 6 h instead of 18 to 24 h).

Other tests for the detection of carbapenemases or carbapenem resistance from blood cultures are available, e.g., PCR, ICTs, or the newly developed EUCAST rapidAST. Using rapidAST, carbapenem resistance can be detected for E. coli or Klebsiella pneumoniae within 4 to 8 h, but no breakpoints are currently available for other *Enterobacterales* species (25). Molecular assays or ICTs only detect the carbapenemases that are targeted, e.g., usually the four or five most common ones (7, 9). However, the identification of the carbapenemase by PCR or ICT has the advantage that treatment can be more rapidly adapted, e.g., ceftazidime-avibactam can be used in most infections by KPC or OXA-48-like producers.

The decision to perform any of the tested assays (or alternative assays such as PCR or ICT) largely depends on the epidemiology and on individual risk factors. In countries with high prevalence (e.g., Italy, Greece or India), the additional information about carbapenemase production will likely be useful and help in clinical decision making. In a low-endemicity setting such as in Germany or the northern European countries with CPE prevalence of <1%, the tests will likely not be cost effective and could be reserved only for patients with risk factors, e.g., patients that are known to be colonized with a CPE or those that have been transferred from a high-risk region.

In this study, we improved current methods for the detection of carbapenemases from blood cultures and systematically compared four different assays on a large set of molecularly characterized CPE isolates. The tests assessed in this study are easy to perform and can detect many different carbapenemases, as they are based on the hydrolysis of a carbapenem. Our study has some limitations. Not all different carbapenemase variants could be tested. Nonduplicate clinical isolates from different years, wards, and centers were used for this evaluation. However, molecular typing was not performed for all isolates, and so clonality of some isolates cannot be fully excluded. Additionally, spiked blood cultures were used, which might not reflect the different conditions present in clinical blood cultures (e.g., different inoculum or incubation time or presence of antibiotics) and could therefore lead to different results compared to those with clinical samples. Nevertheless, we challenged the assays with isolates of 11 different species and 16 different carbapenemases, including many weakly hydrolyzing variants. All assays performed well and could help to optimize therapy early in bloodstream infections by CPE. The protocols can be easily implemented in any clinical microbiology laboratory. Further tests are required to assess the performance of the assays on clinical samples.

## Supplementary Material

Supplemental file 1
