# Supplementary material for: Systematic Comparison of Four Methods for Detection of Carbapenemase-Producing Enterobacterales Directly from Blood Cultures
Source: J Clin Microbiol. 2019 Oct 23;57(11):e00709-19. doi: 10.1128/JCM.00709-19 (PMC6813004; doi:10.1128/JCM.00709-19)
Supplement: Supplemental file 1 [file JCM.00709-19-s0001.pdf]

Systematic comparison of four methods for the detection of carbapenemase-producing Enterobacterales (CPE) directly from blood cultures

Maria Meier and Axel Hamprecht

Supplemental Material

Table S1: Test results of carbapenemase positive isolates

Carbapenemase	Species	Test results<br>β-Carba	 bcCarba NP	 NeoRapid CARB	 bcCIM
**Ambler Class D (n=34)**					
OXA-48 (n=25)	*C. freundii* (n=2)	+	+	+	+
	E. coli (n=14)	+	+	+	+
	E. cloacae (n=2)	+	+	+	+
	K. pneumoniae (n=7)	+	+	+	+
OXA-162 (n=2)	*E. coli* (n=1)	+	+	+	+
	K. pneumoniae (n=1)	+	+	+	+
OXA-181 (n=2)	*E. coli* (n=1)	+	+	+	+
	K. pneumoniae (n=1)	+	+	+	+
OXA-204 (n=1)	*K. pneumoniae* (n=1)	+	+	+	+
OXA-232 (n=2)	*E. coli* (n=1)	+	+	+	+
	K. pneumoniae (n=1)	+	+	+	+
OXA-244 (n=2)	*K. pneumoniae* (n=1)	+	+	-	+
	E. coli (n=1)	+	-	+	+
**Ambler Class A (n=18)**					
KPC-2 (n=17)	*C. brakii* (n=1)	+	+	+	+
	C. freundii (n=1)	+	+	+	+
	K. pneumoniae (n=15)	+	+	+	+
KPC-3 (n=1)	*K. pneumoniae* (n=1)	+	+	+	+
**Ambler Class B (n=50)**					
NDM-1 (n=19)	*E. coli* (n=4)	+	+	+	+
	E. cloacae (n=3)	+	+	+	+
	K. pneumoniae (n=8)	+	+	+	+
	P. mirabilis (n=1)	+	+	+	+
	P. stuartii (n=1)	+	+	+	+
	R. ornithinolytica (n=1)	+	+	+	+
	S. marcescens (n=1)	+	+	+	+
NDM-7 (n=1)	*E. coli* (n=1)	+	+	+	+
VIM-1 (n=18)	*C. freundii* (n=12)	+	+	+	+
	E. coli (n=2)	+	+	+	+
	E. cloacae (n=2)	+	+	+	+
	K. pneumoniae (n=2)	+	+	+	+
VIM-2 (n=3)	*C. freundii* (n=3)	+	+	+	+
VIM-4 (n=2)	*C. freundii* (n=1)	+	+	+	+
	E. cloacae (n=1)	+	+	+	+
VIM-27 (n=1)	*E. cloacae* (n=1)	+	+	+	+
VIM-39 (n=1)	*E. asburiae* (n=1)	+	+	+	+
GIM-1 (n=5)	*E. cloacae* (n=4)	+	+	+	+
	K. oxytoca (n=1)	+	+	+	+
**Multiple Carbapenemases (n=2)**					
NDM-1/OXA-232 (n=1)	*K. pneumoniae* (n=1)	+	+	+	+
NDM-5/OXA-181 (n=1)	*E. coli* (n=1)	+	+	+	+

+ indicates a positive, - indicates a negative test result.

Table S2: Test results of carbapenemase negative isolates

β-lactamase	Species	Test results			
		β-Carba	bcCarba NP	NeoRapid CARB	bcCIM
AmpC (n=2)	*C. freundii* (n=1)	-	-	-	-
	E. coli (n=1)	-	-	-	-
CTX-M-1 (n=4)	*E. coli* (n=4)	-	-	-	-
CTX-M-1/TEM-1 (n=10)	*E. coli* (n=10)	- (n=9)	-	-	-
		+ (n=1)	-	-	-
CTX-M-2/TEM-1 (n=1)	*E. coli* (n=1)	-	-	-	-
CTX-M-3/TEM-1 (n=2)	*C. freundii* (n=1)	-	-	-	-
	K. oxytoca (n=1)	+	+	+	-
CTX-M-9 (n=2)	*E. coli* (n=1)	-	-	-	-
	K. oxytoca (n=1)	+	+	-	-
CTX-M-9/TEM-1 (n=2)	*E. coli* (n=2)	-	-	-	-
CTX-M-9/TEM-1/SHV-1 (n=1)	*K. pneumoniae* (n=1)	-	-	-	-
CTX-M-9/SHV-ESBL (238S+240K) (n=1)	*E. coli* (n=1)	-	-	-	-
CTX-M-15 (n=22)	*E. coli* (n=21)	-	-	- (n=20)	-
		-	-	+ (n=1)	-
	K. pneumoniae (n=1)	-	-	-	-
CTX-M-15/TEM-1 (n=8)	*E. coli* (n=8)	-	-	-	-
CTX-M-15/SHV-1 (n=2)	*K. pneumoniae* (n=2)	-	+	-	-
		-	-	+	-
CTX-M-15/TEM-1/SHV-1 (n=10)	*K. pneumoniae* (n=10)	-	-	- (n=8)	-
		-	-	+ (n=1)	-
		-	-	+ (n=1)	-
CTX-M-15/TEM-1/SHV-ESBL (283S) (n=1)	*K. pneumoniae* (n=1)	-	-	-	-
CTX-M-27 (n=2)	*E. coli* (n=2)	-	-	- (n=1)	-
		-	-	+ (n=1)	-
SHV-ESBL (238S)/TEM-1 (n=1)	*K. pneumoniae* (n=1)	-	-	-	-
SHV-ESBL (238S+240K)/TEM-1 (n=5)	*K. pneumoniae* (n=2)	-	-	-	-
	E. cloacae (n=3)	-	-	-	-
TEM-ESBL (104K+238S) (n=2)	*E. coli* (n=2)	-	-	-	-
K1 hyperproduction (n=1)	*K. oxytoca* (n=1)	+	+	+	-
none (n=2)	*E. coli (n=2)*	-	-	-	-

+ indicates a positive, - indicates a negative test result.

Table S3: Detailed characteristics of isolates with false-positive or false-negative carbapenemase results

		Test results				Minimum Inhibitory Concentration (MIC)		
**Result/β-lactamase**	**Species**	**β-Carba**	**bcCarba NP**	**NeoRapid CARB**	**bcCIM**	**Ertapenem**	**Imipenem**	**Meropenem**
false-negative								
OXA-244	*E. coli*	+	-	+	+	2	0.5	0.25
OXA-244	*K. pneumoniae*	+	+	-	+	32	32	32
false-positive								
CTX-M-9	*K. oxytoca*	+	+	-	-	0.5	0.25	0.25
CTX-M-15	*E. coli*	-	-	+	-	0.06	0.25	0.06
CTX-M-27	*E. coli*	-	-	+	-	0.06	0.25	3
CTX-M-1/TEM-1	*E. coli*	+	-	-	-	0.06	0.5	0.06
CTX-M-3/TEM-1	*K. oxytoca*	+	+	+	-	0.03	0.5	0.06
CTX-M-15/SHV-1/porin loss	*K. pneumoniae*	-	-	+	-	1	0.5	0.25
CTX-M-15/SHV-1	*K. pneumoniae*	-	+	-	-	0.06	0.25	0.06
CTX-M-15/TEM-1/SHV-1	*K. pneumoniae*	-	-	+	-	0.12	0.12	0.06
CTX-M-15/TEM-1/SHV-1	*K. pneumoniae*	-	-	+	-	0.5	0.5	0.06
Hyper K1	*K. oxytoca*	+	+	+	-	0.06	0.25	0.06

Molecular Characterization of carbapenemase-negative isolates

Expression of $bla_{CTX\text{-}M\text{-}1\ group}$, $bla_{CTX\text{-}M\text{-}2\ group}$, $bla_{CTX\text{-}M\text{-}9\ group}$, bla_{TEM} and bla_{SHV} was first assessed using the microarray Check-MDR CT101 (Check-Points, Wageningen, Netherlands). Isolates which were positive for $bla_{CTX\text{-}M\text{-}1\ group}$, $bla_{CTX\text{-}M\text{-}2\ group}$, $bla_{CTX\text{-}M\text{-}9\ group}$ in the first step were further characterized by sequencing the open reading frame using primers listed in table S4.

Table S4: Molecular characterization of ESBL positive isolates

Primer	Sequence 5'-3'	Amplicon [bp]	Reference
CTX-M-1 F	ATG GTT AAA AAA TCA CTG CG		(1)
ORF477_R1	AAA ACA CGG TGG CTA TCA AC	1059	(2)
CTX -M-1 R	TTA CAA ACC GTY GGT GAC	876	(1)
CTX-M-2 F	ATG ATG ACT CAG AGC ATT CG		(3)
CTX-M-2 R	TGG GTT ACG ATT TTC GCC GC	866	(3)
CTX-M-9 F	ATG GTG ACA AAG AGA GTG CAA C		(1)
CTX-M-9 R	TTA CAG CCC TTC GGC GAT G	876	(1)

REFERENCES

1. **Batchelor M, Hopkins K, Threlfall EJ, Clifton-Hadley FA, Stallwood AD, Davies RH, Liebana E.** 2005. bla(CTX-M) genes in clinical Salmonella isolates recovered from humans in England and Wales from 1992 to 2003. Antimicrob Agents Chemother **49:**1319-1322.
2. **Lucena Baeza L, Pfennigwerth N, Hamprecht A.** 2019. Rapid and easy detection of carbapenemases in Enterobacterales in the routine laboratory using the new GenePOC Carba/Revogene Carba C assay. J Clin Microbiol doi:10.1128/JCM.00597-19.
3. **Eckert C, Gautier V, Saladin-Allard M, Hidri N, Verdet C, Ould-Hocine Z, Barnaud G, Delisle F, Rossier A, Lambert T, Philippon A, Arlet G.** 2004. Dissemination of CTX-M-type beta-lactamases among clinical isolates of Enterobacteriaceae in Paris, France. Antimicrob Agents Chemother **48:**1249-1255.
